# Physiotherapy Care of Patients with Coronavirus Disease 2019 (COVID-19) - A Brazilian Experience

**DOI:** 10.6061/clinics/2020/e2017

**Published:** 2020-06-16

**Authors:** Renato Fraga Righetti, Mirian Akemi Onoue, Flavia Vanessa Aurea Politi, Débora Trigo Teixeira, Patricia Nery de Souza, Claudia Seiko Kondo, Eliana Vieira Moderno, Igor Gutierrez Moraes, Ana Lígia Vasconcellos Maida, Laerte Pastore, Felipe Duarte Silva, Christina May Moran de Brito, Wania Regina Mollo Baia, Wellington Pereira Yamaguti

**Affiliations:** Hospital Sirio-Libanes, Sao Paulo, SP, BR.

**Keywords:** COVID-19, Physiotherapy, Mechanical Ventilation, Oxygen Therapy, Severe Acute Respiratory Syndrome Coronavirus 2

## Abstract

Some patients with coronavirus disease (COVID-19) present with severe acute respiratory syndrome, which causes multiple organ dysfunction, besides dysfunction of the respiratory system, that requires invasive procedures. On the basis of the opinions of front-line experts and a review of the relevant literature on several topics, we proposed clinical practice recommendations on the following aspects for physiotherapists facing challenges in treating patients and containing virus spread: 1. personal protective equipment, 2. conventional chest physiotherapy, 3. exercise and early mobilization, 4. oxygen therapy, 5. nebulizer treatment, 6. non-invasive ventilation and high-flow nasal oxygen, 7. endotracheal intubation, 8. protective mechanical ventilation, 9. management of mechanical ventilation in severe and refractory cases of hypoxemia, 10. prone positioning, 11. cuff pressure, 12. tube and nasotracheal suction, 13. humidifier use for ventilated patients, 14. methods of weaning ventilated patients and extubation, and 15. equipment and hand hygiene. These recommendations can serve as clinical practice guidelines for physiotherapists. This article details the development of guidelines on these aspects for physiotherapy of patients with COVID-19.

## INTRODUCTION

The current outbreak of coronavirus disease 2019 (COVID-19) originated in the Hubei Province of the People’s Republic of China (1,2), and on March 11, 2020, it was declared a pandemic by the World Health Organization Emergency Committee (2).

The most common symptoms include fever (89%), cough (68%), fatigue (38%), sputum production (34%), and shortness of breath (19%) (3). A considerable proportion of the population with COVID-19 will not require hospitalization as the patients present a mild or uncomplicated form of the disease with a favorable prognosis. However, older patients and those with chronic underlying conditions can develop severe illness and present complications such as acute respiratory disease syndrome (ARDS), sepsis, septic shock, and kidney and cardiac failure, which require treatment in an intensive care unit (ICU) with invasive support (4). Approximately 14% of patients develop a severe form of COVID-19, requiring hospitalization, and 5% require admission to an ICU (5).

Physiotherapists are recognized in several countries as professionals working in primary and tertiary care (6-8) who play a fundamental role in multi-professional teams providing ventilatory support during the acute illness phase and rehabilitation interventions thereafter to promote functionality (9,10).

This paper describes the different actions and practices adopted by the Rehabilitation Service of Hospital Sírio-Libanês (São Paulo, Brazil) to face the challenges in treating and containing the spread of COVID-19. Issues pertaining to clinical practice in the adult hospital setting were identified on the basis of the experience and opinions of front-line experts as well as a review of the relevant literature.

To provide the maximum level of care and ensure staff protection, recommendations were developed regarding protective equipment, conventional chest physiotherapy, exercise and early mobilization, oxygen therapy, nebulizer treatment, non-invasive ventilation and high-flow nasal oxygen, endotracheal intubation, protective mechanical ventilation, management of mechanical ventilation in severe and refractory cases of hypoxemia, prone positioning, cuff pressure, tube and nasotracheal suction, humidifier use for ventilated patients, methods of weaning ventilated patients, and equipment and hand hygiene.

## PERSONAL PROTECTIVE EQUIPMENT (PPE)

Several procedures performed by physiotherapists may generate aerosols and droplets, which are sources of lung and respiratory pathogens. These procedures include non-invasive ventilation, high-flow oxygenation, endotracheal intubation, airway tracheostomy and endotracheal tube suction, cardiopulmonary resuscitation, high-frequency oscillatory ventilation, chest physiotherapy, prone patient positioning, disconnection of the ventilator, administration of nebulized treatment, and sputum induction (11-14). Furthermore, severe acute respiratory syndrome coronavirus 2 can remain in the air for hours and on surfaces of various materials for days upon aerosolization, with risks of possible human infection (15). However, when aerosol-generating procedures cannot be avoided, they should be performed in a negative-pressure room. In the absence of negative-pressure rooms, the procedures must be performed in a room with closed doors and open windows; with minimum number of qualified professionals to perform the procedures; with appropriate PPE; and avoiding the presence other people (16,17). Therefore, physiotherapists must adopt protective measures to avoid aerosol exposure and for contact isolation by using adequate PPE, namely, surgical caps, safety goggles, face shields, N95 masks or equivalent, gowns, and gloves (17-19).

## CONVENTIONAL CHEST PHYSIOTHERAPY

Currently, no evidence exists indicating that conventional chest physiotherapy changes the course of COVID-19 in the acute phase of the disease in patients with hypoxemic respiratory failure and dry cough. However, some patients with productive cough may benefit from bronchial hygiene maneuvers and techniques that stimulate coughing (20,21). Patients with a mild form of the disease should be instructed to perform breathing exercises independently. Patients with moderate and severe conditions should be constantly monitored for pulmonary disease (22). In these cases, physiotherapists should contact the patient only for respiratory and pulmonary assessments, especially during orotracheal intubation and oxygen supplementation and for patients who are candidates for non-invasive ventilation or high-flow oxygen administration (22,23). The professional exposure time should be the minimum necessary for evaluation and assistance (22,24).

## EXERCISE AND EARLY MOBILIZATION

Patients usually present with a debilitated physical condition because of the disease, which reduces their exercise capacity, especially when they present with fever, dyspnea, myalgia, and fatigue (20); the debilitated physical condition can also be a result of prolonged mechanical ventilation and immobilization. Hospitalized patients, even those with moderate disease severity, can spend weeks in hospital isolation, with a significant decrease in their activity levels, and are thus prone to a reduction in their muscle strength and cardiorespiratory capacity (25). Therefore, patients in the acute phase with mild disease should be encouraged to perform light-intensity exercises to maintain minimal functional capacity. The exercises can be tailored for maintenance of a Borg rating of <3 (on a 10-point scale) (22). Although there are no studies specific to patients with COVID-19, classically critical patients who underwent early mobilization showed a reduction in delirium and duration of mechanical ventilation (26); thus, early mobilization should be started as soon as possible, as long as the patient presents suitable clinical conditions (27). This mobilization can include neuromuscular stimulation, therapeutic exercises, and early verticalization (28-31).

## OXYGEN THERAPY

The prevalence of hypoxic respiratory failure in adults with COVID-19 is 19%; thus, oxygen therapy represents a major treatment intervention for patients with severe pulmonary dysfunction (2,32). Adults with COVID-19 should be started on supplemental oxygen if the peripheral oxygen saturation (SpO_2_) is <93% and maintained oxygen saturation is no higher than 96% (23). Mechanical ventilation may be necessary in cases of respiratory failure refractory to oxygen therapy (2,23).

The interfaces used for oxygen supplementation can generate aerosols. Therefore, health care workers should take adequate precautions and wear proper PPE when providing respiratory support to patients with COVID-19 complicated by respiratory failure (19,33). Oxygen humidification should not be used (34). The prescription of moisturizers such as self-applied nasal sodium chloride gel may be suggested for complications such as dryness of the upper airways or epistaxis. The oxygen supply device should be changed if these complications persist.

**Figure 1:**
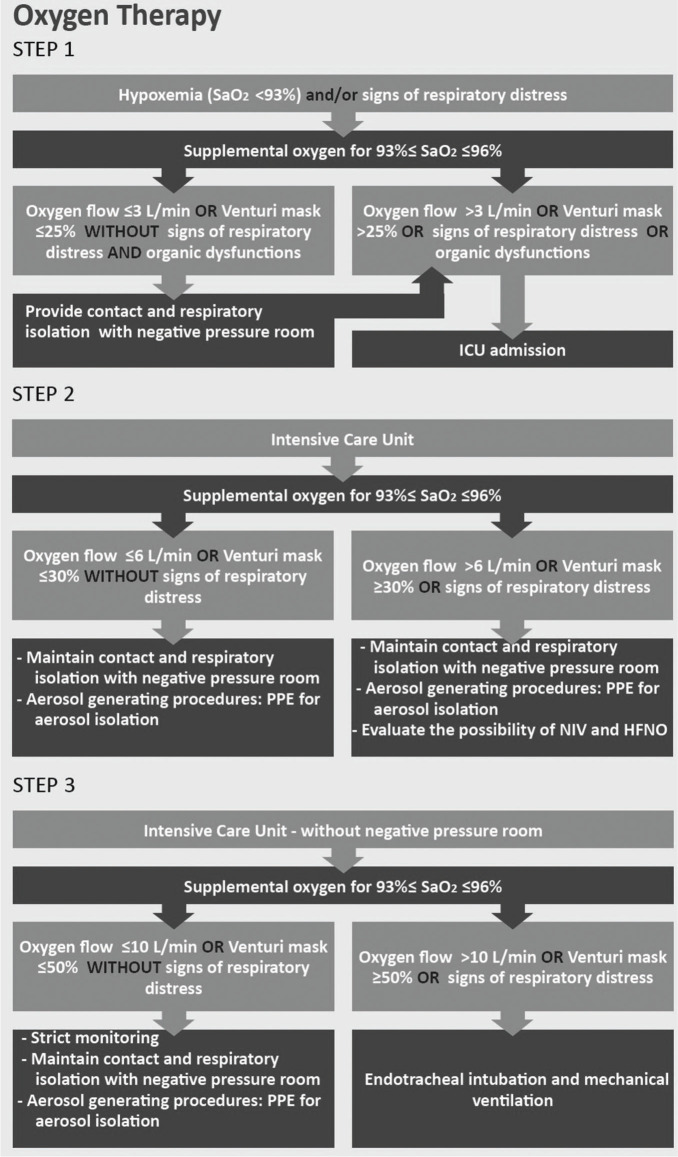
Proposal for oxygen therapy and early transfer to intensive care units for patients with respiratory distress and hypoxemia based on Surviving Sepsis Campaign: Guidelines on the Management of Critically Ill Adults with Coronavirus Disease 2019 (COVID-19). SaO2: arterial oxygen saturation; ICU: intensive care unit; PPE: Personal protective equipment; NIV: non-invasive ventilation; HFNO: high-flow nasal oxygen.

Figure 1 shows our institutional proposal for oxygen therapy and early transfer to the ICU for patients with respiratory distress and hypoxemia on the basis of the Surviving Sepsis Campaign: Guidelines on the Management of Critically Ill Adults with Coronavirus Disease 2019 (COVID-19).

## NEBULIZER TREATMENT

All forms of nebulization (including inhalation) are potential aerosol generators and should be avoided (2,35). Bronchodilators should be administered with metering units (puff or spray) in an air chamber/spacer (2).

## NON-INVASIVE VENTILATION AND HIGH-FLOW NASAL OXYGEN

For the treatment of acute hypoxemic respiratory failure, the use of high-flow nasal oxygen is suggested over conventional oxygen therapy and non-invasive positive pressure ventilation (36-38). If high-flow nasal oxygen is not available, a trial of non-invasive ventilation is suggested (39). An experiment in a human model showed that non-invasive ventilation or high-flow nasal oxygen, when well applied with an optimal fit, resulted in minimal aerosolization of exhaled air (40). However, the specific models of masks and interfaces tested in the study are not universally used in all hospitals. Therefore, to avoid potential harm, we recommend using adequate precautions and PPE and discourage the use of this procedure if an airborne infection isolation room is unavailable (2,16). Monitoring for worsening respiratory status and subsequent early intubation is recommended (39).

Patient candidates for non-invasive ventilation admitted to the ICU in negative-pressure rooms must be ventilated with positive end-expiratory pressure (PEEP) ≥8 cmH_2_O, support pressure for a tidal volume (TV) ≤8 mL/kg of the predicted weight, and fraction of inspired oxygen (FiO_2_) to maintain SaO_2_ >92%. Facial or full-face masks must be used during application of the ventilator. Devices with double branches for ventilation are indicated in these cases, with a heat moisture exchange filter (HMEF) between the face mask and the device and another high-efficiency particulate arrestance (HEPA) filter on the exhalation outlet of the ventilator. For high-flow oxygen, a flow rate of 40 to 50 L/min should be maintained, and FiO_2_ to maintain SaO_2_ >92% should be started.

**Figure 2:**
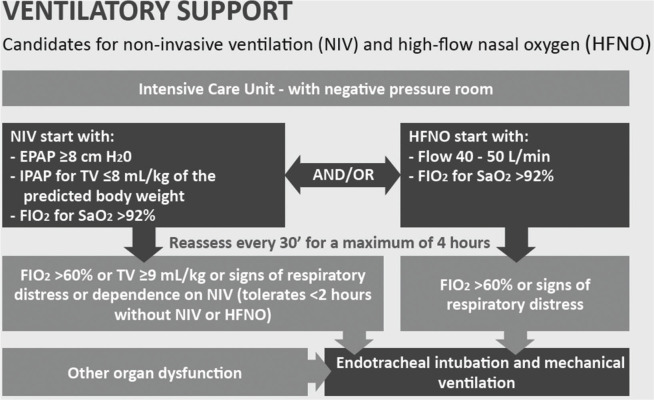
Proposal for non-invasive ventilation and high-flow nasal oxygen for patients with COVID-19. NIV: non-invasive ventilation; EPAP: expiratory positive airway pressure; IPAP: inspiratory positive airway pressure; TV: tidal volume; FiO2: fraction of inspired oxygen; HFNO: high-flow nasal oxygen; SaO2: arterial oxygen saturation.

The criteria for orotracheal intubation and invasive mechanical ventilation are FiO_2_ >60% in non-invasive ventilation or TV ≥9 mL/kg or inability to tolerate <2 hours without non-invasive ventilation or presence of other organic dysfunctions. For high-flow oxygen, the criteria for orotracheal intubation are FiO_2_ >60% or signs of respiratory distress, or other organic dysfunctions. It is important to reassess the patient after 30 to 60 minutes; if there is no improvement or if there is worsening of ventilatory parameters, endotracheal intubation and invasive mechanical ventilation should be considered (Figure 2) (2,23,36-38).

## ENDOTRACHEAL INTUBATION

When aerosol-generating procedures are required, they are recommended to be performed in a negative-pressure room and with the use of appropriate PPE (16). Only the professionals needed to perform orotracheal intubation should remain in the room.

**Figure 3:**
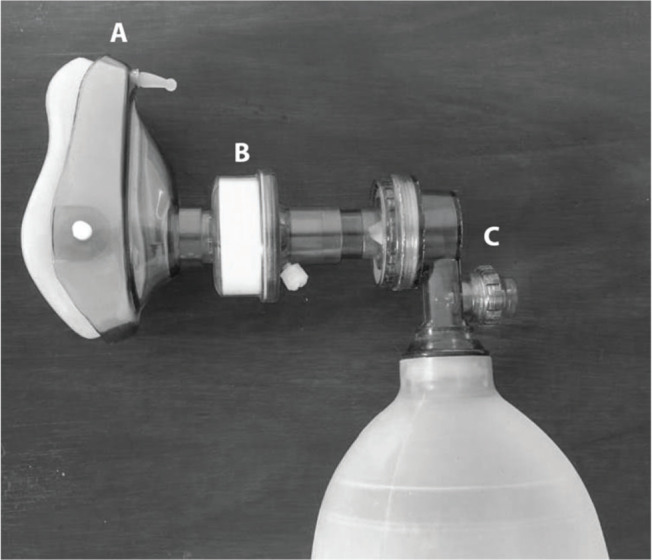
Bag-Valve-Mask device: (A) face mask, (B) heat moisture exchange filter, and (C) Bag-Valve-Mask.

Patients with COVID‐19 are at risk of a rapid decrease in arterial oxygen levels; therefore, effective pre-oxygenation is mandatory. Patients must be administered a sufficient oxygen flow to maintain SpO_2_ >93%, and intubation should be performed with a rapid sequence of induction and intubation. Pre-oxygenation with a non-rebreather mask with the lowest possible airflow to maintain effective oxygenation (SpO_2_ >93%) (41) is required. It is also important to avoid assisted ventilation with the Bag-Valve-Mask device or the use of a supraglottic device because of the potential for aerosolization and contamination of health workers. However, if necessary, we suggest adding a filter between the simple respirator and the Bag-Valve-Mask or artificial airway during use (Figure 3) to reduce the spread of the virus in the patient's airway to the indoor air (19).

After orotracheal intubation, checking the proper positioning of the orotracheal tube and inflating the cuff are recommended. The patient can then be connected to the ventilator associated with the HMEF and with a HEPA filter in the expiratory valve of the mechanical ventilator. These filters can filter bacteria and viruses and reduce room contamination (2,41-43). Airway interventions must be carried out by experienced individuals. After each procedure, appropriate hand hygiene is required (41).

## PROTECTIVE MECHANICAL VENTILATION

Invasive mechanical mode volume-controlled ventilation (in the presence of neuromuscular block or the absence of inspiratory effort) or pressure-controlled ventilation (in the absence of neuromuscular block and mild respiratory effort and asynchrony) should be performed with lower TVs (4 to 6 mL/kg predicted body weight) and lower inspiratory pressures, reaching a plateau pressure (Pplat) of <28-30 cmH_2_O (44). The PEEP must be as high as possible to maintain the driving pressure (Pplat − PEEP) as low as possible (<15 cmH_2_O) and SpO_2_ 88-95% (44,45). Moreover, disconnection from the invasive mechanical ventilator must be avoided to prevent loss of PEEP and consequent atelectasis.

## MANAGEMENT OF MECHANICAL VENTILATION IN SEVERE AND REFRACTORY CASES OF HYPOXEMIA

For patients with PaO_2_/FiO_2_ <150 and an inability to maintain protective ventilation or with the presence of asynchrony or severe hypercapnia (pH <7.25), we suggest sedation and continuous neuromuscular block to reduce respiratory drive and maintain protective ventilation. The multidisciplinary team can discuss the following: 1. prone positioning; 2. alveolar recruitment maneuvers and PEEP adjustment for better pulmonary compliance; 3. recruitment in the prone position for patients who responded to the supine recruitment maneuver; 4. nitric oxide administration in cases with a clinical history of “cor pulmonale” or as a recruitment maneuver for hypoxemia; and 5. extracorporeal membrane oxygenation (ECMO) (2,44,46,47).

## PRONE POSITION

Prone ventilation for 12 to 16 hours a day is recommended in adult patients with severe ARDS (PaO_2_/FiO_2_ <150), (2,44). It is strongly recommended for adult patients with severe ARDS but requires sufficient human resources and knowledge to be performed safely. Protocols and videos are available in the study by Guérin et al., 2013. A satisfactory response is defined as a patient achieving an increase of 10 mmHg in PaO_2_ or an increase of 20 mmHg in the PaO_2_/FiO_2_ ratio. Prone positioning should be repeated when a PaO_2_/FiO_2_ ratio <150 mmHg is observed after 6 hours in the supine position. PaO_2_/FiO_2_ reductions of 20% in the supine position should be considered criteria for interrupting the prone position after two consecutive attempts at pronation or hemodynamic instability (48,49).

## CUFF PRESSURE

Invasive mechanical ventilation is a risk factor for aerosols (50). Therefore, it is important to maintain a cuff pressure between 20 and 30 cmH_2_O or 25 and 35 mmHg, with sufficient pressure to prevent leakage and aerosol spread (51). We suggest cuff measurement either at every shift or at least daily (51).

## TUBE AND NASOTRACHEAL SUCTION

Suction of the artificial airway because of ventilator disconnection must be avoided so that there is no loss of pressure in the respiratory system, atelectasis, or spread of aerosols in the room. The use of a closed suction system in all cases of intubation and invasive mechanical ventilation is recommended (2,50). In situations requiring open suction, we suggest the use of the “stand by” mode of the mechanical ventilator to minimize the spread of aerosols. Nasotracheal suction should be performed with careful evaluation by the physiotherapist because of the generation of aerosols. To perform these procedures, the use of proper PPE is recommended. Whenever possible, this procedure should be performed in a negative-pressure room.

## HUMIDIFIERS FOR VENTILATED PATIENTS

Heat and moisture exchangers or heated humidifiers are more effective in preventing complications such as airway blockages and pneumonia in adults who receive invasive mechanical ventilation (52). Therefore, patients with COVID-19 should use devices that humidify and filter their inhaled and exhaled air, respectively. Thus, HMEF is more suitable for the humidification of exchanged air as it also has filtering capacity for viruses and bacteria, thus reducing air contamination. Additional protection can be provided by placing a HEPA filter on the exhalation valve of the mechanical ventilator. The use of heated humidifiers is discouraged in these patients (43).

## WEANING FROM MECHANICAL VENTILATION AND EXTUBATION

All patients must be evaluated daily regarding the eligibility criteria for the spontaneous breathing test, considering adequate oxygenation: PaO2/FiO2>200 with PEEP ≤5-7 cmH2O, hemodynamic stability with low and stabilized doses or without vasopressor drug infusion, an adequate level of consciousness (easily awake or wakened), and adequate cough and secretion management with the presence of a cough reflex during closed aspiration (53,54).

To wean patients with COVID-19 from mechanical invasive ventilation, we recommend the use of the pressure support ventilation (PSV) mode for spontaneous breathing tests. The use of the T-tube method should be avoided as it can increase aerosolization (43). Table 1 shows the parameters suggested for the spontaneous breathing test in PSV (A), success criteria (B), and failure criteria (C) (55-60). The cuff leak test should not be performed routinely before extubation because of the risk of aerosolization. However, its use should be considered for the clinical suspicion of upper airway edema or the presence of risk factors for post-extubation stridor (61).

Patients who pass the spontaneous breathing test should preferably be extubated in a negative-pressure room or in respiratory isolation. Physiotherapists and other health professionals present in the environment during extubation must follow PPE aerosol isolation precautions. During the procedure, extra care must be taken during extubation, including keeping the HMEF and closed endotracheal suction (e. g. Trach-Care®) connected to the endotracheal tube when deflating the cuff. The endotracheal tube should be removed as gently as possible to avoid vigorous manipulation and coughing. If it is necessary to stimulate the patient's cough, the patient should be instructed to adopt cough etiquette. The tube must be discarded in the infectious waste collector. In the ICU, the availability of a professional with experience in intubation is always recommended during the extubation of patients diagnosed with COVID-19, in case rapid reintubation is necessary. The rate of reintubation of these patients should be as low as possible; therefore, we recommend that the decision regarding the patient's extubation be rigorously discussed within the multidisciplinary team (62,63).

**Figure 4:**
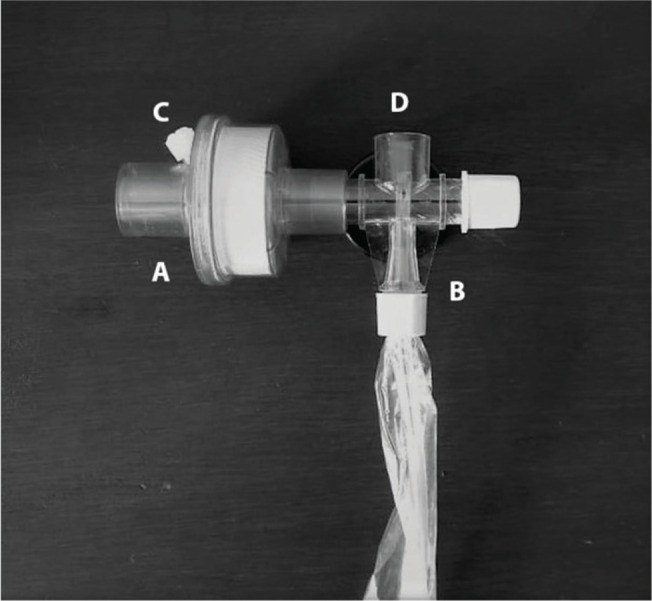
Spontaneous breathing test device for tracheostomized patients: (A) heat moisture exchange filter (HMEF), (B) closed endotracheal suction (Trach-care), (C) site for connection of oxygen therapy in HMEF. (D) connection for the tracheostomy.

Tracheostomy may be indicated for patients who consecutively fail to wean or with long periods of intubation. Tracheostomy is considered a high-risk procedure for the formation of aerosols. Weaning patients using tracheostomy masks (e.g., Trach-Vent® and T-tube) is not recommended for patients with COVID-19. Rather, for spontaneous breathing training periods, the use of HMEF connected to Trach-Care® (Figure 4), with oxygen supplementation directly in the HMEF to maintain SpO2 between 93 and 96%, is recommended. If aspiration is required during the spontaneous breathing test, the closed suction system must be used. We emphasize that the use of HMEF to wean tracheostomized patients requires constant assessment of clinical signs of discomfort or instability. Spontaneous breathing time should be progressive as patients improve breathing performance and resistance (64).

## EQUIPMENT AND HAND HYGIENE

The cleaning of the equipment with 70% alcohol or chlorine-based substances is recommended immediately after use. Health workers should wash their hands frequently, especially after contact with infected people or their environment (2).

## CONCLUSION

COVID-19 is a new disease that presents challenges to inpatient care. These recommendations can serve as clinical practice guidelines for physiotherapists. Physiotherapy plays a fundamental role throughout patient hospitalization. However, the hospital physiotherapy team must be well-oriented regarding specific care to both reduce infection risk and provide the best patient care. The Appendix section presents cards from our institution, Hospital Sírio-Libanês, in English and Portuguese languages for the respiratory management of patients with suspected or confirmed COVID-19 infection. [Fig f05][Fig f06][Fig f07][Fig f08][Fig f09][Fig f10]

## APPENDIX

**Figure 5 f05:**
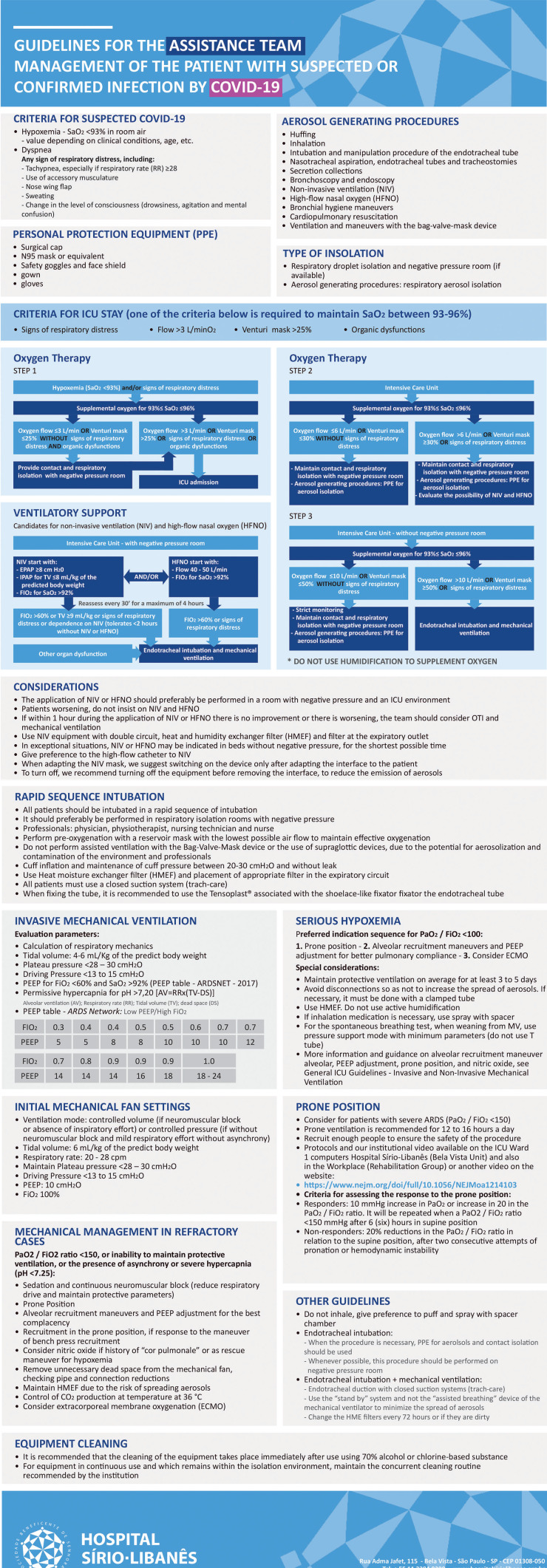


**Figure 6 f06:**
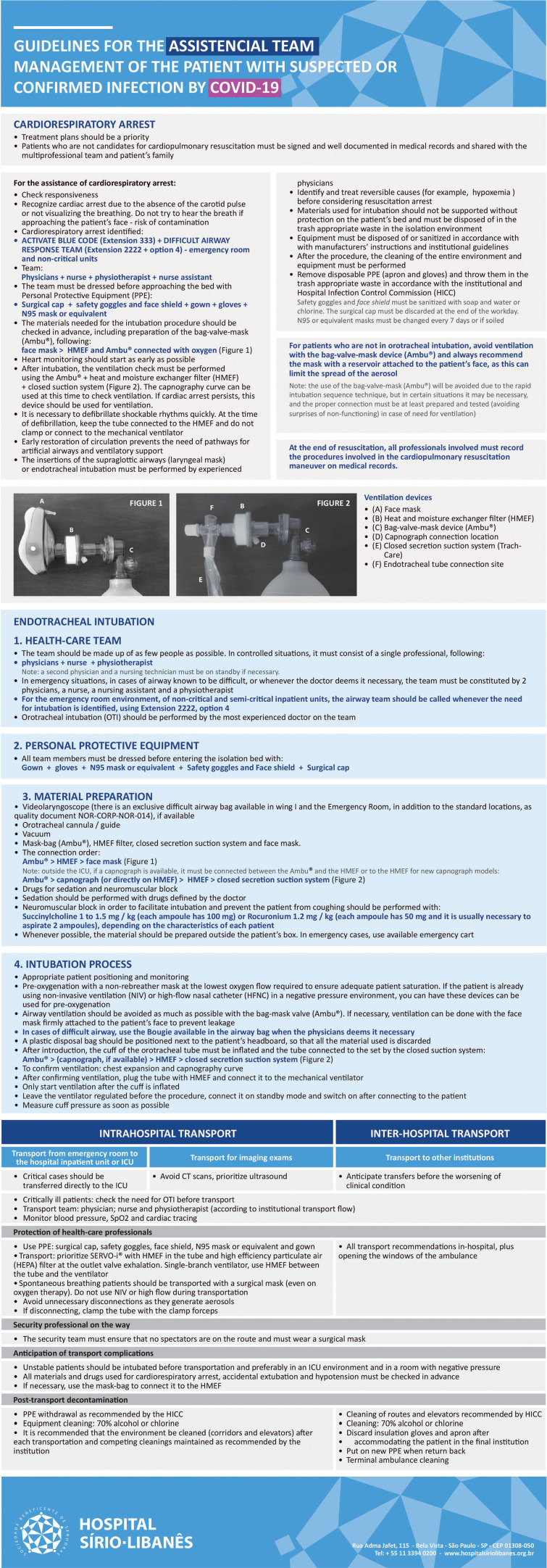


**Figure 7 f07:**
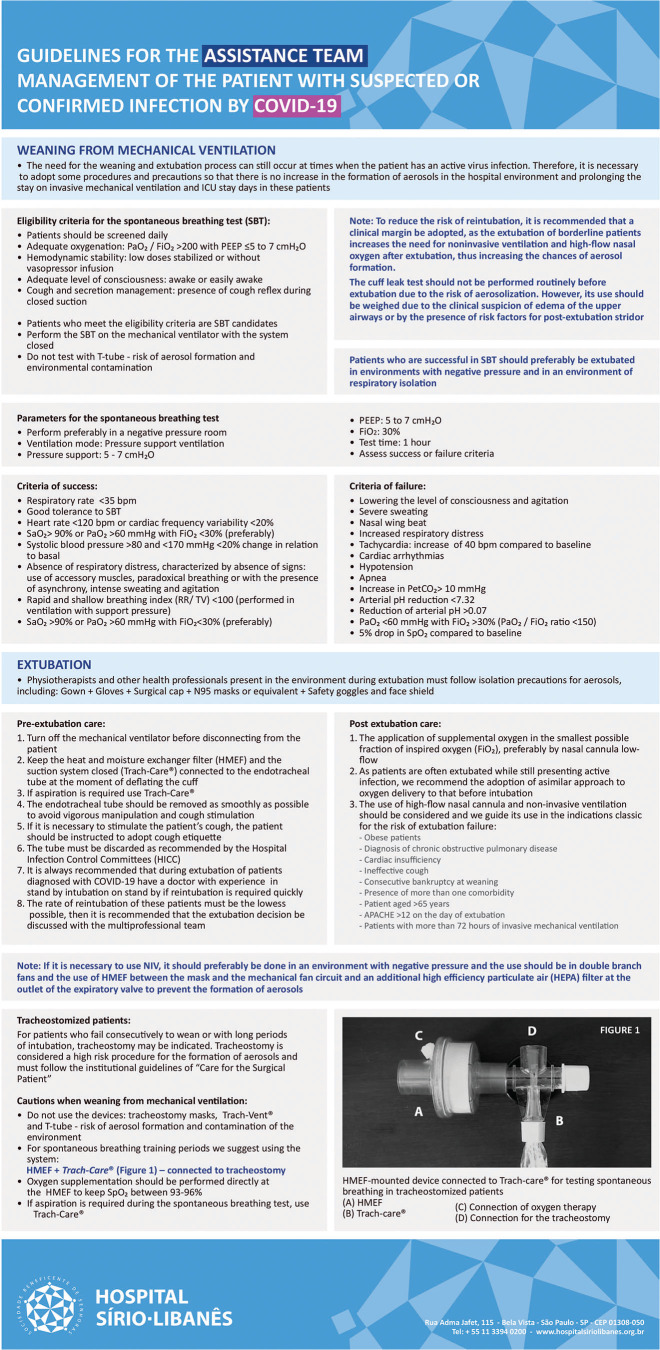


**Figure 8 f08:**
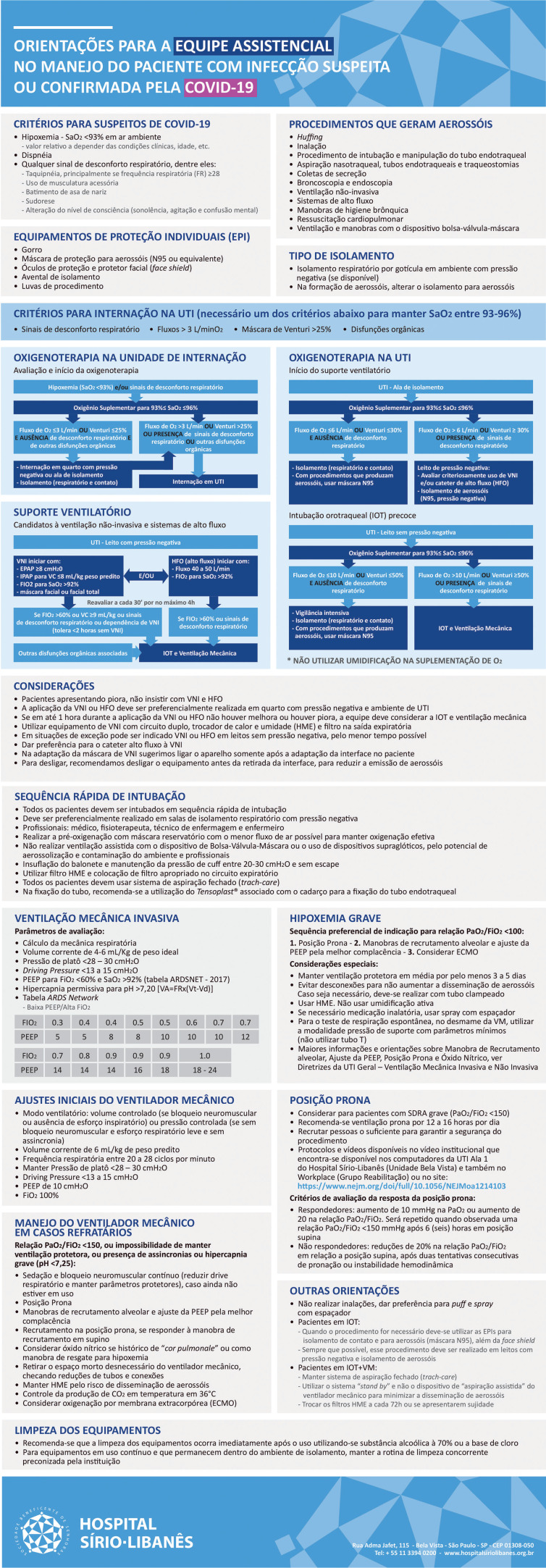


**Figure 9 f09:**
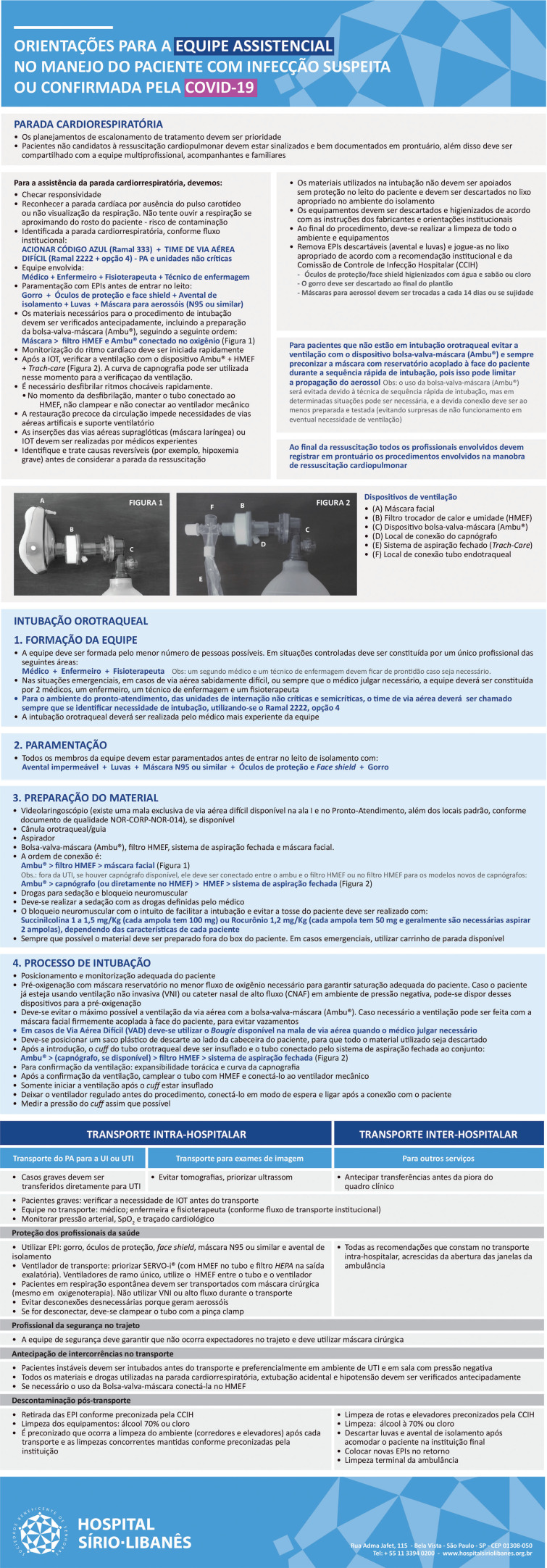


**Figure 10 f10:**
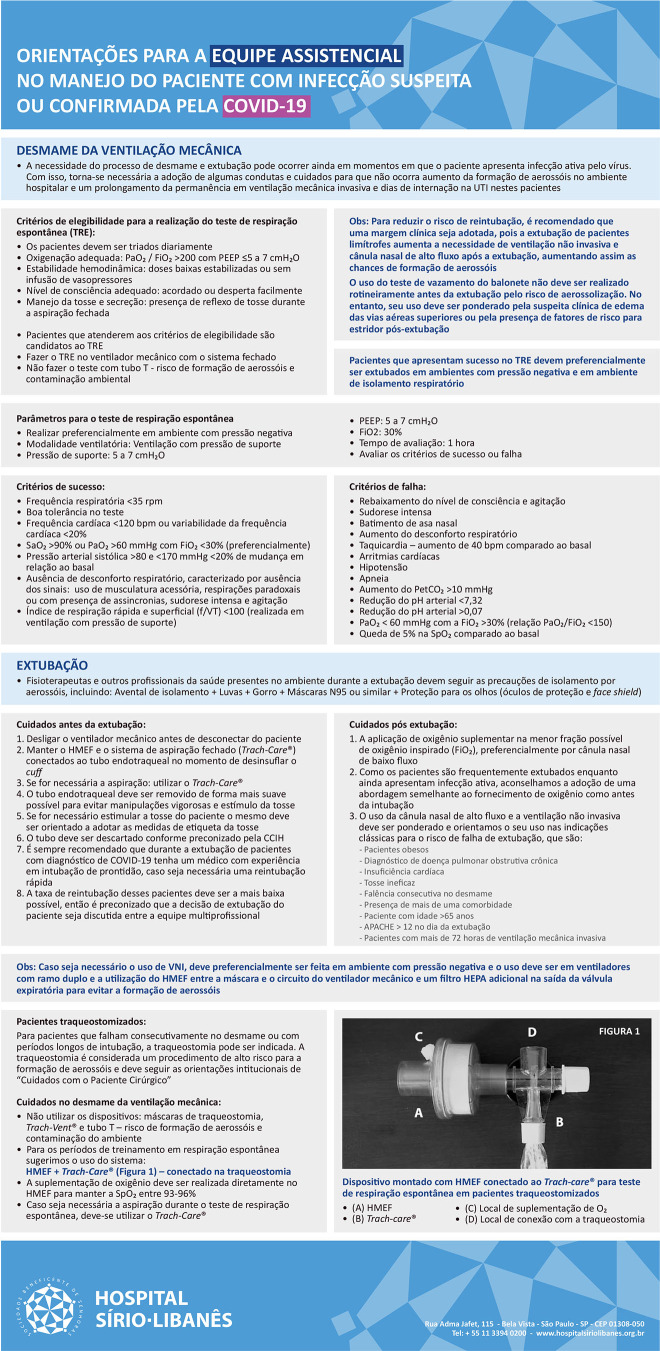


## AUTHOR CONTRIBUTIONS

Righetti RF, Onoue MA, Politi FVA, Teixeira DT, Souza PN, Kondo CS, Moderno EV, Moraes IG, Maida ALV, Pastore Junior L and Silva FD helped in the manuscript design and drafting. Righetti RF and Yamaguti WP were responsible for the study conception and manuscript design and drafting. Brito CMM, Baia WRM and Yamaguti WP are the senior authors who were responsible for study supervision and revision of the final manuscript version. All authors approved the final manuscript version.

## Figures and Tables

**Table 1:** Parameters suggested for the spontaneous breathing test in pressure support ventilation (PSV) (A), success criteria (B), and failure criteria (C).

A
**Spontaneous breathing test parameters**
Mode of ventilation: Presure Support Ventilation
Pressure support: 5 to 7 cmH2O
PEEP: 5 to 7 cmH2O
FiO2: 30%
Test time: 1 hour
B
**Criteria for success**
Respiratory rate <35 bpm
Good tolerance to spontaneous breathing trials
Heart rate <120 per minute or heart rate variability of <20%
SaO2 >90% or PaO2 >60 mmHg with FiO2 <30% (preferably)
Systolic blood pressure >80 and <170 mmHg or <20% change from baseline
No signs of labored breathing or distress
Rapid shallow breathing index <100
C
**Criteria for failure**
Decreased level of consciousness
Nostril flaring
Diaphoresis
Apnea
Tachycardia with increased heart rate >40 per minute
Hypotension
Cardiac arrhythmias
Increasing respiratory effort
Increase of PetCO2 >10 mmHg
Decrease of arterial pH <7.32
Decline in arterial pH >0.07
PaO2 <60 mmHg with FiO2 >30% (PaO2/FiO2 ratio <150)
Fall in SpO2 >5% compared to the basal value

Legends: PEEP: positive end-expiratory pressure; FiO2: fraction of inspired oxygen; bpm: breaths per minute; SaO2: arterial oxygen saturation; PetCO2: end-tidal carbon dioxide pressure; pH: ptential of hydrogen; PaO2: arterial oxygen pressure; SpO2: peripheral oxygen saturation.
